# A single-seed uniform distribution and spreading device for real-time detection of *Ambrosia artemisiifolia* and *Ambrosia trifida* seeds in imported soybeans

**DOI:** 10.3389/fpls.2025.1677883

**Published:** 2025-10-07

**Authors:** Ze Liu, Xi Qiao, Jianwei Qiang, Shuo Zhang, Zhihui Tian, Yujuan Gu, Jun Chen

**Affiliations:** ^1^ College of Mechanical and Electronic Engineering, Northwest A&F University, Yangling, China; ^2^ Shenzhen Branch, Guangdong Laboratory of Lingnan Modern Agriculture, Agricultural Genomics Institute at Shenzhen, Chinese Academy of Agricultural Sciences, Shenzhen, China; ^3^ Genome Analysis Laboratory of the Ministry of Agriculture and Rural Affairs, Agricultural Genomics Institute at Shenzhen, Chinese Academy of Agricultural Sciences, Shenzhen, China; ^4^ Shaanxi Agricultural Machinery Research Institute Co., Ltd., Xianyang, China; ^5^ Guangzhou Customs District Technology Center, Guangzhou, China

**Keywords:** soybean quarantine, device design, parabolic seed socket, Taguchi experimental design, deep learning

## Abstract

China relies heavily on imported soybeans due to insufficient domestic production, but these imports are often contaminated with quarantine weed seeds such as *A. artemisiifolia* and *A. trifida*. The introduction of these species poses serious ecological risks, highlighting the urgent need for reliable real-time detection methods. In this study, a single-seed uniform distribution and spreading device was designed to minimise occlusion and ensure consistent seed visibility. The device integrates a parabolic seed-socket distribution unit with an embedded system. After seeds were arranged in a single layer on a conveyor belt, a detection camera captured images that were processed by the YOLO_P2 model for seed recognition and counting. Device performance was optimised using the Taguchi experimental design, and evaluated with signal-to-noise ratio, mean, and variance. Experimental analysis revealed that the speeds of the seed-spreading roller and conveyor motor were the most significant factors affecting distribution uniformity. Validation experiments showed that the optimised system achieved detection accuracies of 95.73% for A. trifida and 94.41% for A. artemisiifolia, with an average processing time of 7.6 minutes per sample. These results demonstrate that the proposed device provides a practical, cost-effective solution for quarantine inspection, combining high-throughput capability with real-time performance to support ecological protection efforts.

## 1 Introduction

Soybean is a highly nutritious crop widely used in various consumer products and traded globally in large volumes (de Lima et al., 2018). Due to their high volume and broad distribution, soybean shipments are particularly vulnerable to contamination by quarantine weed seeds. The spread of these seeds leads to serious economic (including direct damages and the costs of mitigation and prevention), health (e.g., globally emerging zoonotic diseases), and environmental (e.g., decline in native biodiversity) issues (Jay et al., 2003; Hall, 2011; Baider and Florens, 2011). According to the InvaCost Database, the global mean annual cost associated with invasive alien species may reach USD 162.7 billion (Diagne et al., 2021). Additionally, variation in quarantine standards across countries results in differing criteria for determining sample acceptability (Davies and Sheley, 2007), posing major challenges to the effective implementation of quarantine procedures. These issues highlight the need for effective measures to detect, control, and manage the spread of these harmful organisms (Meyerson and Reaser, 2002; Magarey et al., 2009). Consequently, the development of a rapid and accurate method for detecting quarantine weed seeds is critically important.

Currently, the identification of quarantine weed seeds largely relies on manual techniques such as protein electrophoresis, DNA barcoding, and gas chromatography (Huang et al., 2015; Lei et al., 2017; Whitehurst et al., 2020). However, these methods are often costly and time-consuming, making them unsuitable for large-scale applications (Rahman and Cho, 2016; ElMasry et al., 2019). With advances in electronics and microprocessor technology, the development of detection devices has offered new approaches for the identification of quarantine weed seeds (Thakuria and Erkinbaev, 2024; Zhao et al., 2025). Several devices and systems have been developed for seed sorting and quality assessment. Zhao et al. (2021) developed a real-time detection system for comprehensive surface inspection of soybeans, in which pneumatic actuators rotated the samples for multi-angle imaging. An enhanced MobileNetV2 model was applied, achieving an accuracy of 98.87%. Similarly, Wang et al. (2023) proposed an online cottonseed classification device using a rotary disc and fixed partitions for single-seed positioning. Dual cameras captured images of falling seeds, and a YOLOv5-based model classified them as intact, damaged, or moldy, achieving an accuracy of 99.6%. Despite their reliable detection capabilities, the high construction and maintenance costs have limited their practical application in agriculture. Furthermore, since these devices are generally designed for specific seed types, their effectiveness is reduced in quarantine inspections where seed diversity is high. Most existing devices perform detection after singulation. While manageable for small quantities, high-throughput quarantine inspections make efficient singulation difficult. These challenges underscore the importance of developing a real-time, high-throughput detection system that is cost-effective and adaptable to a variety of seed types.

Deep learning models have demonstrated exceptional performance in object recognition tasks (Vega−Castellote et al., 2025; Cao et al., 2025), and YOLOv8n is both lightweight and accurate (Chen et al., 2025; Zheng et al., 2024). Improvements made by Ma et al. (2024) and Liu et al. (2024) boosted model accuracies to 91.4% and 84.12%, respectively. These studies show that deep learning models can effectively detect various target types, supporting the recognition and counting of imported soybean and quarantine weed seeds. However, performance decreases significantly in cases of target occlusion. While model enhancements can improve accuracy, they typically involve significant computational demands and only offer marginal gains (Íñiguez et al., 2024). Moreover, detecting small targets among variably sized seeds remains difficult (Zhang et al., 2025), requiring targeted adjustments to meet the accuracy standards of quarantine inspection.

Currently, no commercially available intelligent detection devices are specifically designed for quarantine weed seeds. To address this gap, the present study developed a real-time, high-throughput detection device capable of cost-effective acquisition of seed image datasets. The design maximizes the number of seeds captured per image, avoids mutual occlusion, and accommodates practical operating conditions. The device integrates a seed distribution mechanism and an embedded system to achieve high-quality image acquisition, recognition, and counting. A seed dispenser incorporating parabolic seed sockets was designed to ensure compatibility with a variety of seed types. The Taguchi experimental design method was applied to optimize configuration parameters for efficiently collecting high-quality seed images.

The main objectives of this study were as follows: first, to enable high-throughput acquisition of high-quality seed image datasets, a seed dispenser with parabolic seed sockets was developed. This design ensured uniform distribution of multiple seed types within a single image, maximizing seed quantity and avoiding mutual occlusion. Second, to optimize the quality of seed distribution, the device parameters were experimentally investigated and refined using the Taguchi method, resulting in the optimal configuration. Finally, to achieve real-time detection and improve the accuracy for small targets (specifically *A. artemisiifolia* seeds), targeted modifications were applied to the YOLO_P2 model, significantly enhancing its detection performance.

## 2 Materials and methods

### 2.1 Seed sample preparation

**Figure 1:**
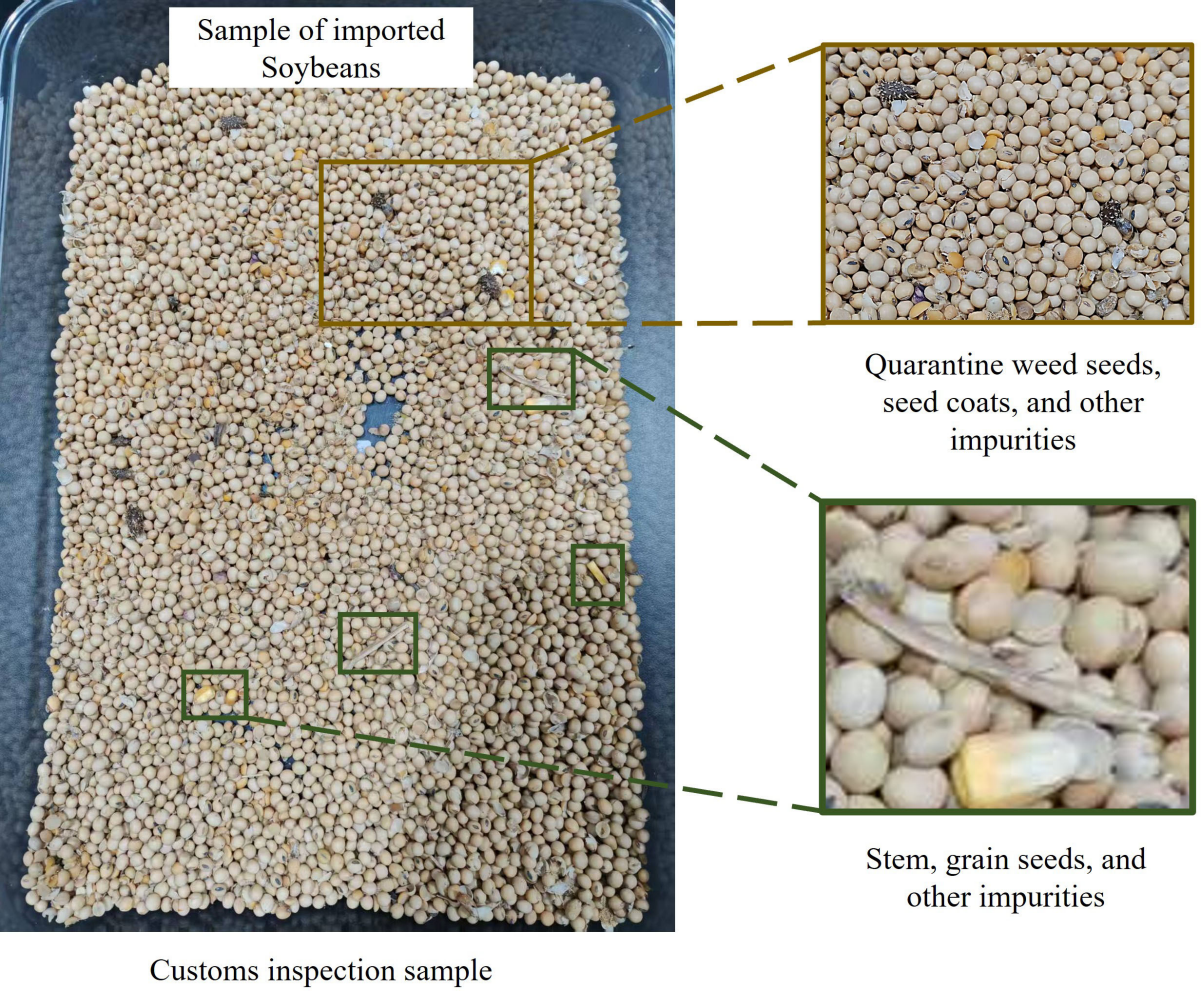
Customs inspection sample.

**Figure 2:**
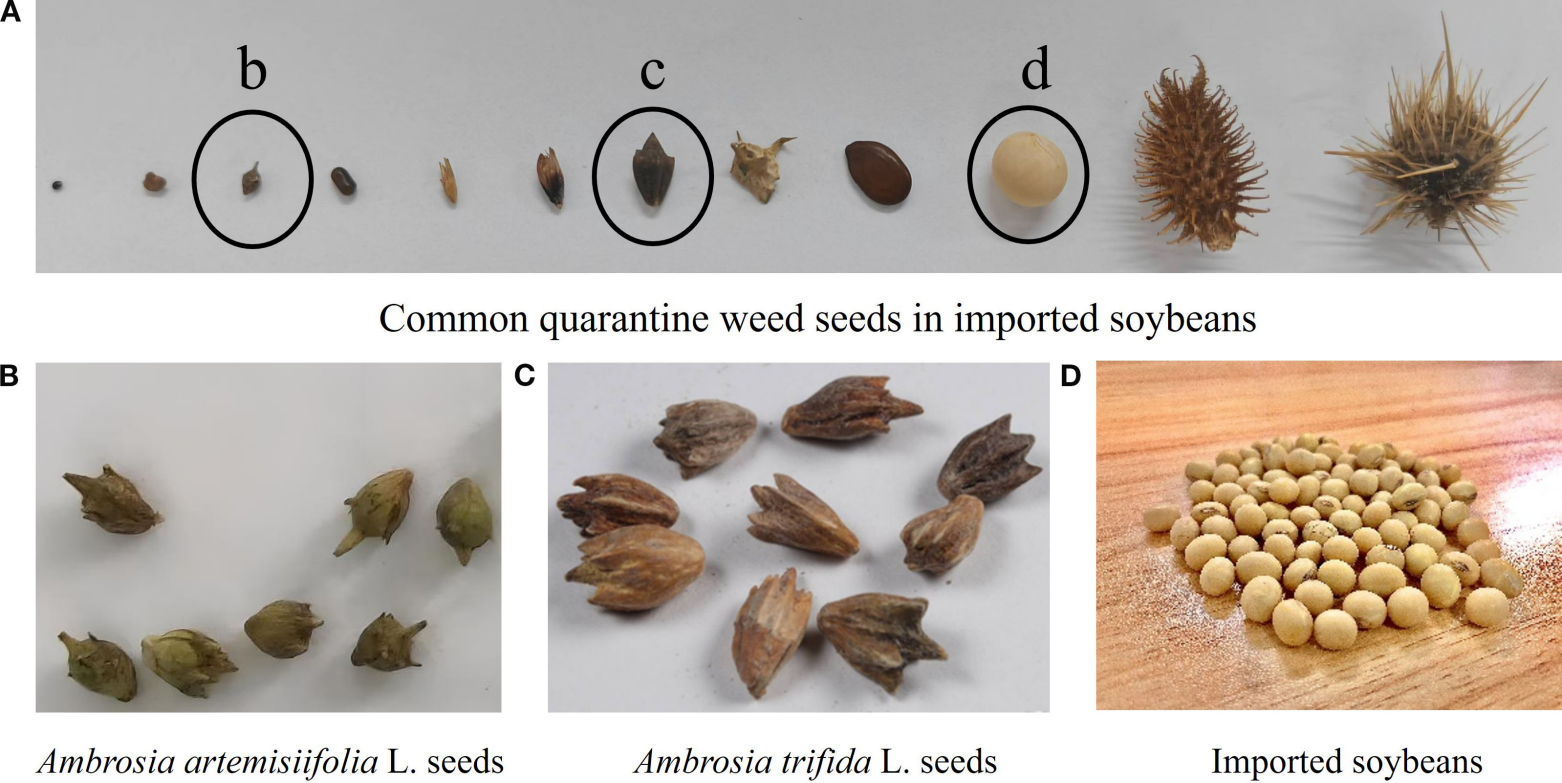
Common quarantine weed seeds and imported soybeans.

As shown in **Figure 1**, the samples submitted to customs for inspection contain various impurities. In addition to quarantine weed seeds, impurities such as seed coats, miscellaneous grain seeds, plant stems, and stones are also present, which poses significant challenges for manual quarantine inspection. Although preliminary screening can remove some impurities that are larger than imported soybeans, including certain quarantine weed seeds, miscellaneous grains, plant stems, and stones, the majority of quarantine weed seeds are smaller than imported soybeans (**Figure 2A**). Although existing studies using deep learning methods are capable of identifying these types of seeds, their accuracy decreases significantly under conditions such as target occlusion or poor lighting. Therefore, the ability to rapidly and continuously acquire high-quality sample image datasets, in which seeds are uniformly arranged and not occluded, remains a major challenge that has yet to be resolved. **Figures 1** and **2** present illustrative images of common impurities in imported soybean samples, which are provided solely for background explanation and were not used in model training, validation, or performance evaluation.

To support the design of the seed dispenser, seed types were first selected. The seed samples included Brazilian soybeans (**Figure 2D**), which represent the primary source of global soybean imports (Ali et al., 2022), along with common quarantine weed seeds found in imported soybeans, as illustrated in **Figure 2**. Larger seeds, such as Xanthium spinosum Linn. and *Solanum rostratum Dunal* (**Figure 2A**), can be removed through preliminary screening. For smaller weed seeds, two representative species, *Ambrosia artemisiifolia* L. (**Figure 2B**) and *Ambrosia trifida* L. (**Figure 2C**), were selected due to their high interception rates in imported soybeans. These seeds were provided by the Agricultural Genomics Institute in Shenzhen, Chinese Academy of Agricultural Sciences, in May 2025. The imported soybeans used in the subsequent experiments had a moisture content of 12%.

**Figure 3:**
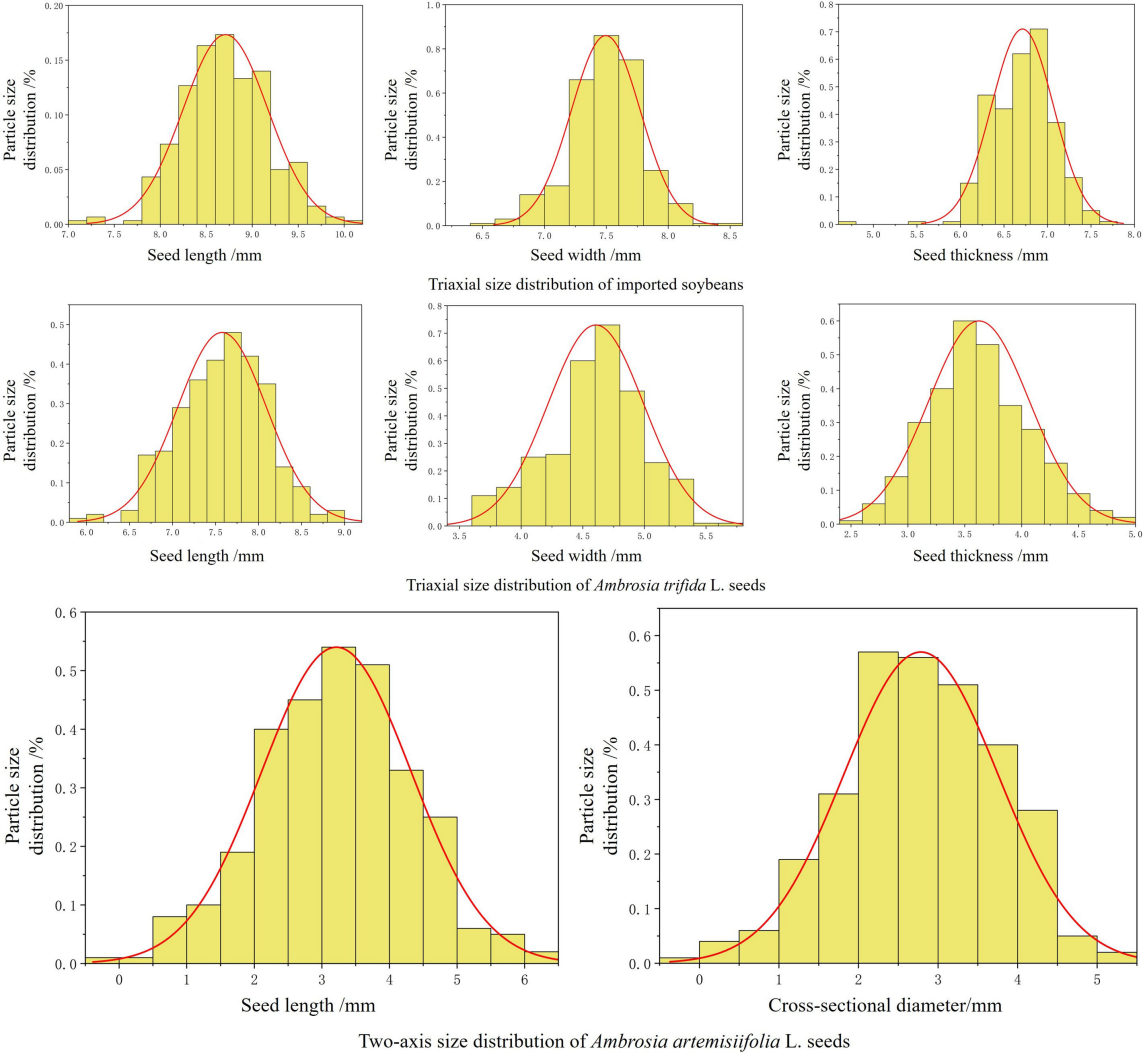
Seed size and particle size distribution.

The dimensions of imported soybeans and two types of quarantine weed seeds were measured. For each seed type, 500 ± 10 samples were randomly selected and measured using a digital vernier caliper with an accuracy of 0.01 mm (DELIXI Electric, Hangzhou, China). The dimensional and particle size distributions are shown in **Figure 3**. The average values were used as design references. The imported soybeans had an average length of 7.65 mm, a width of 6.52 mm, and a thickness of 5.70 mm. *A. trifida* seeds had an average length of 7.57 mm, a width of 4.57 mm, and a thickness of 3.69 mm. Due to the smaller size of *A. artemisiifolia* seeds and the minimal difference between their width and thickness, only the length and cross-sectional diameter were measured, with average values of 3.22 mm and 2.84 mm, respectively.

### 2.2 Single-seed uniform distribution and spreading device

The method of detecting only imported soybeans is not suitable for customs quarantine scenarios, as customs authorities assess sample compliance based on the proportion of quarantine weed seeds present. Therefore, all three types of seeds must be detected and counted. Currently, the specific evaluation criteria adopted by Chinese Customs remain confidential and have not been publicly disclosed. Moreover, there is a lack of research on detection devices specifically designed for identifying quarantine weed seeds in imported bulk agricultural commodities. In response to this gap, the present study developed a real-time, high-throughput, and cost-effective detection device, with the goal of minimizing detection time and maximizing detection accuracy.

**Figure 4:**
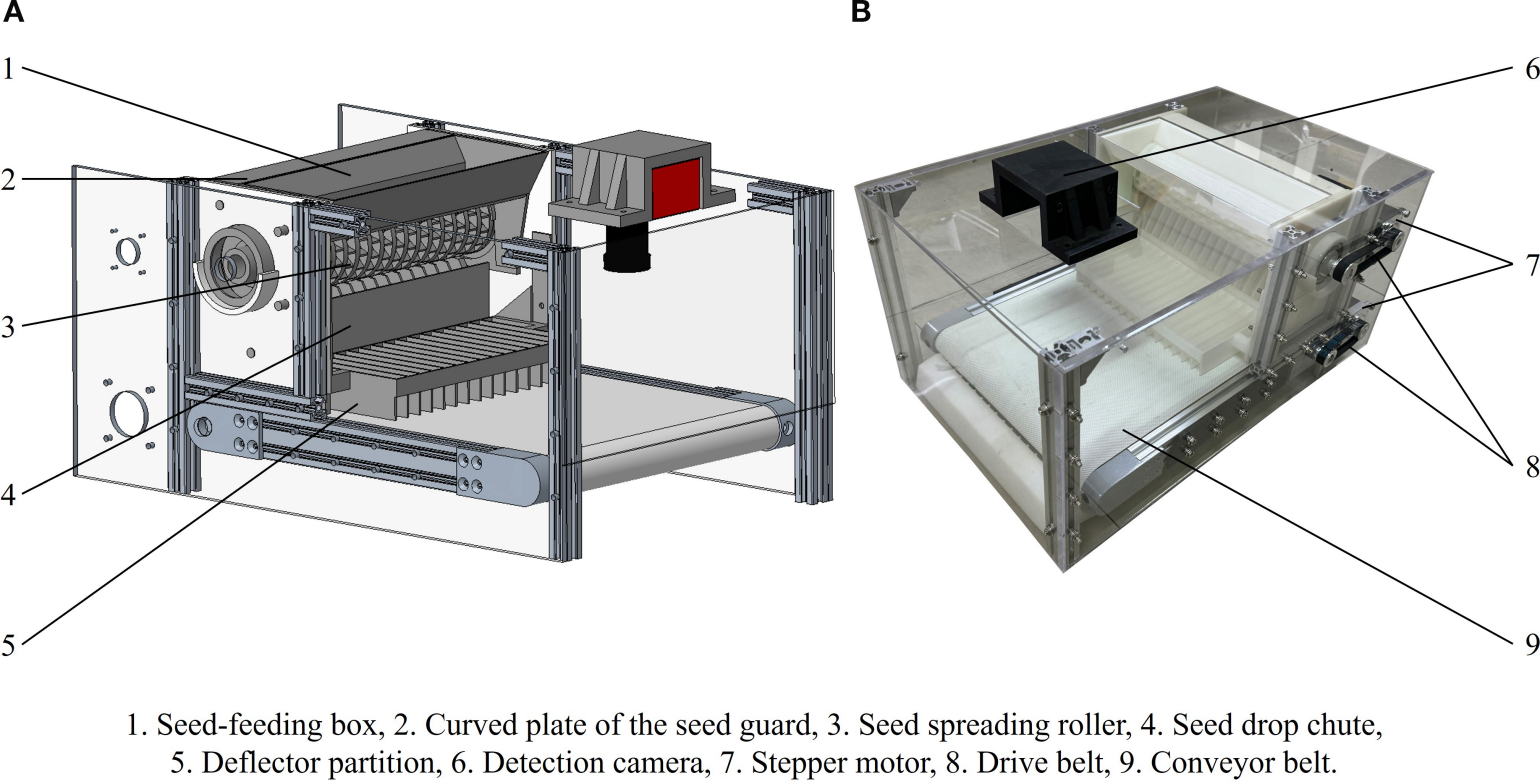
Schematic **(A)** and photograph **(B)** of single-seed uniform distribution and spreading device.

The detection device measures 262 cm in height, 544 cm in length, and 334 cm in width. The single-seed uniform distribution and spreading mechanism consists of a seed dispenser and a conveyor belt, as illustrated in **Figure 4**.

**Figure 5:**
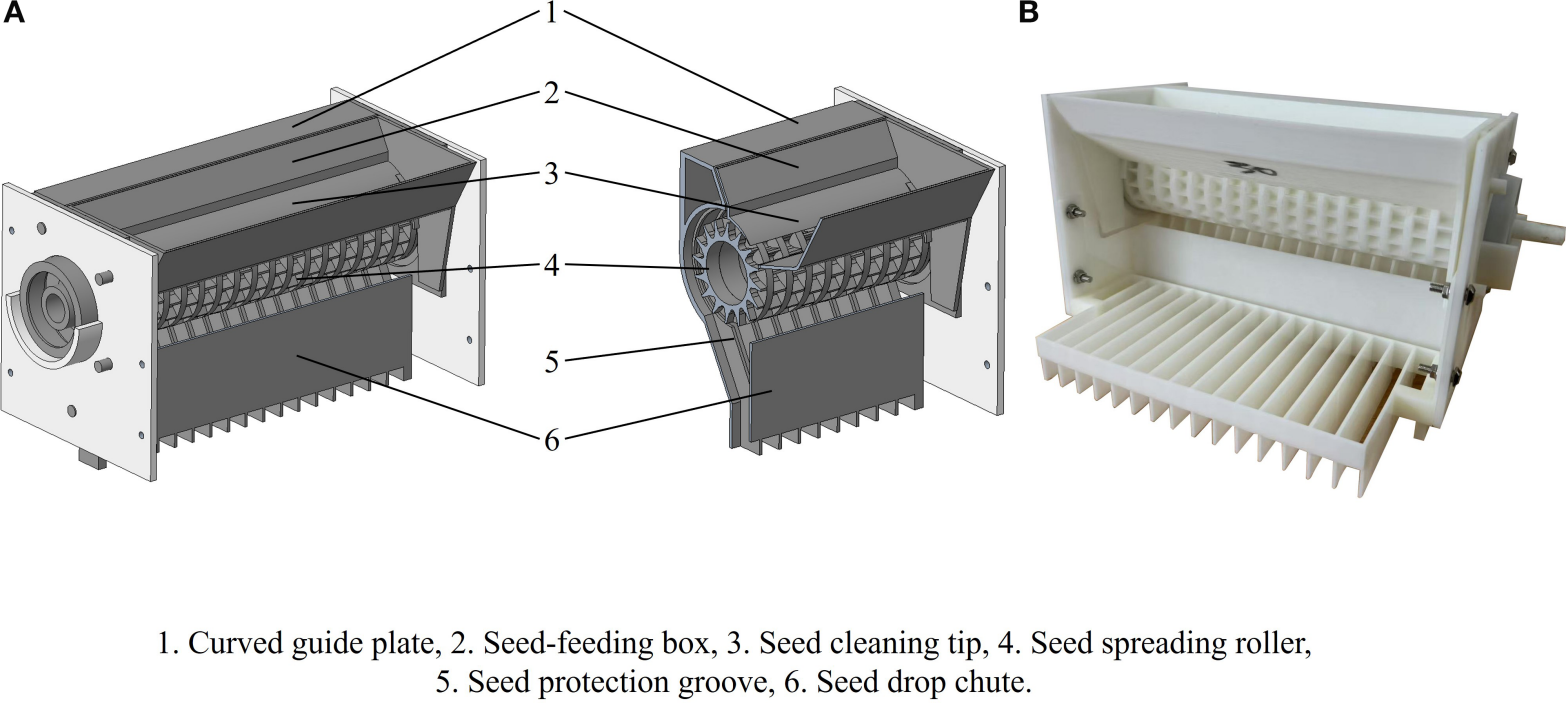
Schematic **(A)** and photograph **(B)** of the seed dispenser.

The seed dispenser comprises a seed feeding box, a seed spreading roller, a curved guide plate, and a seed drop chute, as illustrated in **Figure 5**. The spreading roller is equipped with parabolic seed sockets arranged in 17 staggered columns, with each column offset by 15 degrees. The conveyor module consists of a conveyor belt driven by a stepper motor and features a white, diamond-patterned belt designed to reduce seed bouncing and minimize light reflection. The embedded system is positioned on one side of the detection device. For continuous real-time image acquisition, a detection camera (AOSVI Micro HK830, Shenzhen, China) with a resolution of 3840 × 2160 pixels is mounted at the top of the device. The camera is equipped with a 6 mm adjustable focal length lens and is positioned 20 cm above the detection area. To reduce the impact of ambient light fluctuations on detection accuracy, an LED light source is installed at the top of the detection area.

**Figure 6:**
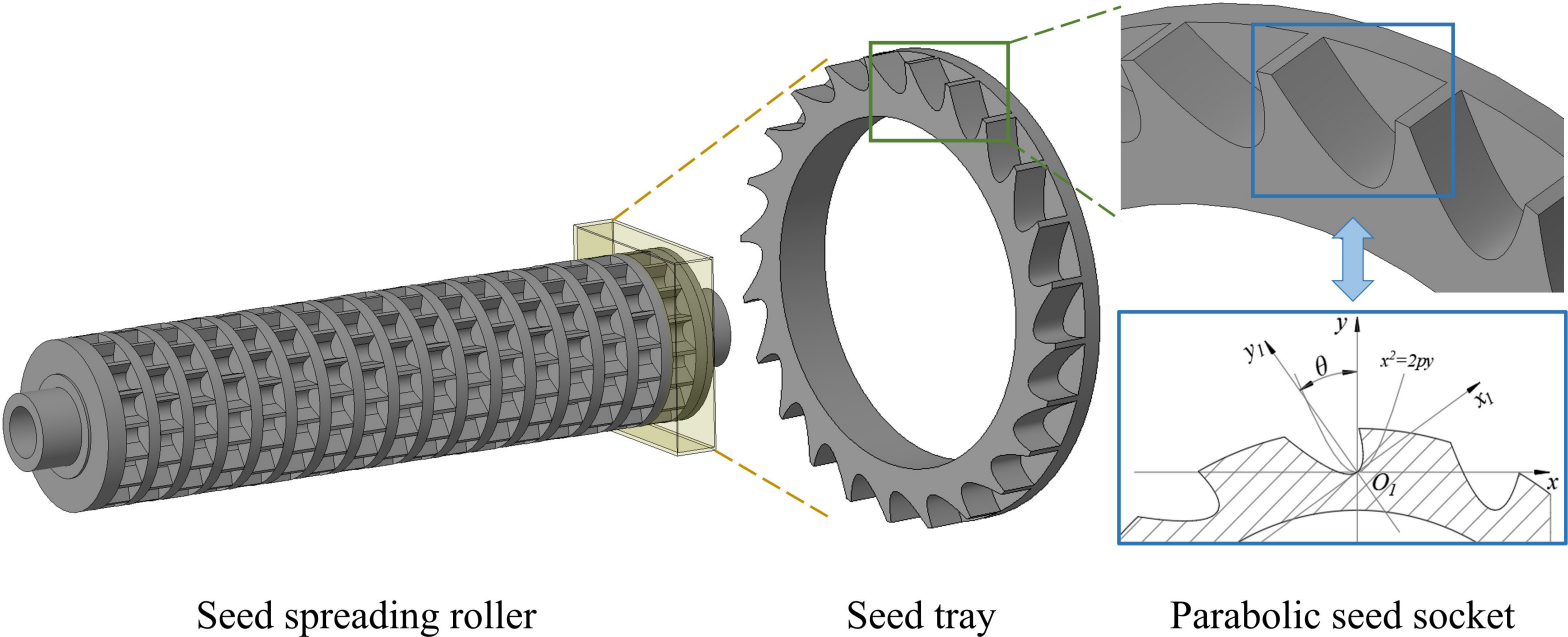
Seed spreading roller with parabolic seed socket.

The seed spreading roller plays a pivotal role in determining the performance of the seed dispenser. Key design parameters of the roller include its diameter, the number of seed sockets, and the socket geometry. Considering the typical diameters used in existing soybean dispensers and the spatial constraints of the overall system, a diameter of 120 mm was selected. Uniform seed distribution depends heavily on the compatibility between the socket shape and the physical characteristics of imported soybeans and quarantine weed seeds. The three seed types, which are imported soybeans, *A. artemisiifolia*, and *A. trifida*, differ significantly in both shape and size. Their proportions are typically unbalanced, with soybeans accounting for the majority of the mixture. To address this issue, a parabolic seed socket was designed based on the equation *x² = 2py*. This shape has a zero slope at the vertex, which allows seeds to naturally decelerate and stabilize within the cavity. The narrowing profile toward the bottom accommodates seeds of different sizes. In addition, the sockets are inclined at a specific angle to improve the efficiency of seed filling, transportation, and discharge. This design reduces filling time and ensures more stable seed retention. The final specifications of the socket include a length of 14 mm, a width of 10 mm, a focal length *p* = 2.98 mm, and a rotation angle *θ* = 32.46°, with 24 sockets arranged on each tray. A schematic diagram of the roller and socket is shown in **Figure 6**.

### 2.3 Real-time quarantine weed seeds detection system

**Figure 7:**
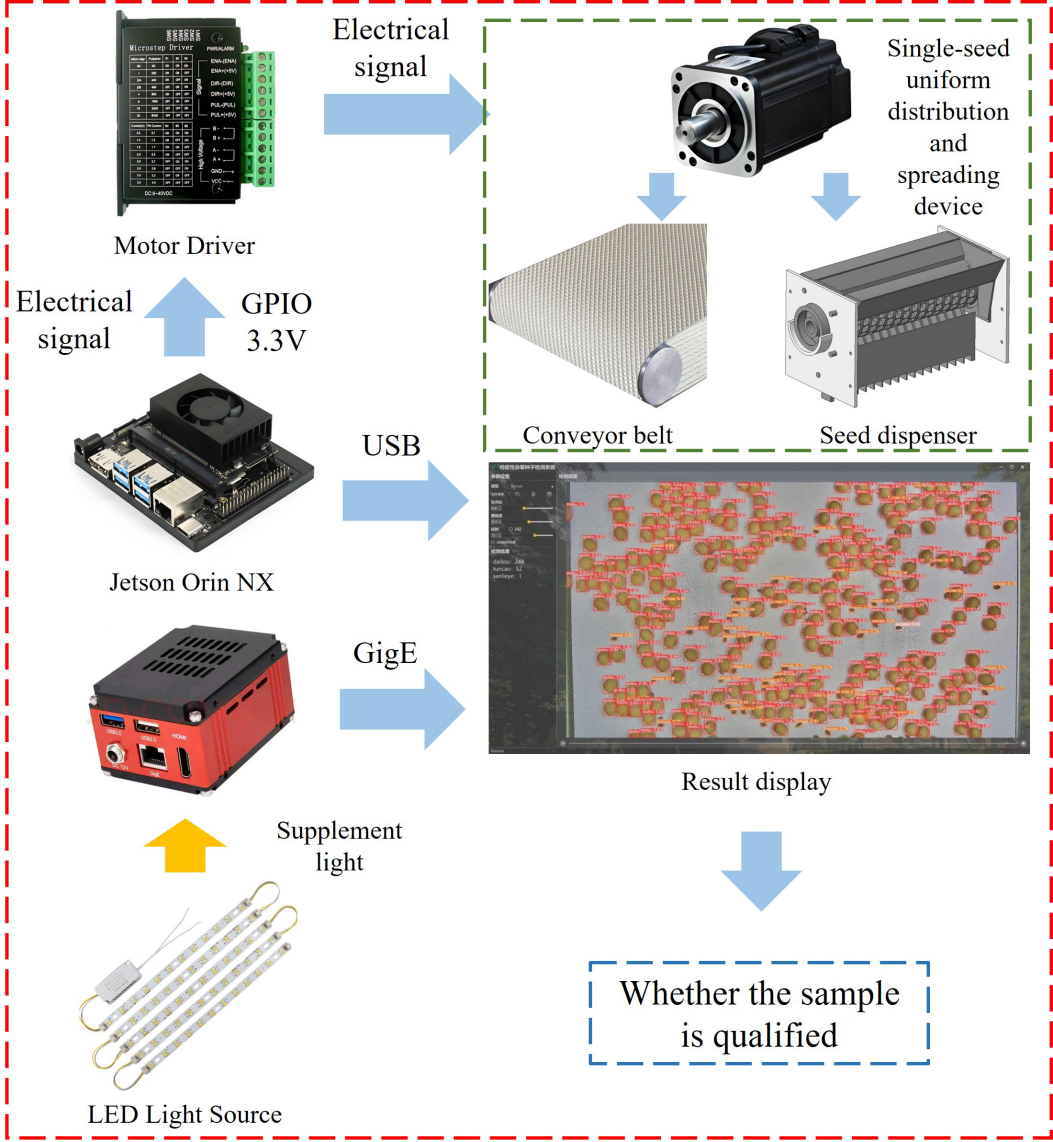
Real-time detection system.

The real-time quarantine weed seed detection system consists of a detection camera, an LED light source, a display screen, and a Jetson Orin NX device. The Jetson Orin NX is responsible for controlling the stepper motors that drive the single-seed uniform distribution and spreading mechanism. It also manages the detection camera to capture and process seed images. The detection workflow and seed counting results are displayed in real time on the screen, as shown in **Figure 7**. During operation, the imported soybeans and quarantine weed seeds are dispensed through the seed dispenser and pass through the seed spreading roller, where they are individually separated into the parabolic seed sockets. The shaped seeds then fall onto the conveyor belt via the curved guide plate of the seed guard. As the seeds move through the detection area, the detection camera captures images for subsequent analysis.

The Jetson Orin NX generates a Pulse Width Modulation (PWM) signal at a designated frequency, which is transmitted through the TB6600 driver and combined with the drive parameters of both the spreading roller and the conveyor stepper motors to maintain a consistent rotational speed. At the same time, the Jetson Orin NX dynamically adjusts the exposure interval of the detection camera based on the speed of the conveyor belt that transports the seeds. This synchronization ensures that all imported soybeans and quarantine weed seeds passing through the detection zone are accurately captured by the camera. As a result, missed detections are avoided and duplicate counts are prevented.

### 2.4 Taguchi experimental design

#### 2.4.1 Experimental setup

Within the image acquisition area, the effectiveness of the seed dispenser in achieving uniform seed spreading may lead to varying degrees of adhesion and occlusion, which significantly affect the detection of quarantine weed seeds. To mitigate this issue, the Taguchi method was adopted to analyze and optimize the parameters influencing the performance of the single-seed uniform distribution and spreading device. Proposed by Japanese scholar Genichi Taguchi, this method combines orthogonal experimental design with Signal-to-Noise Ratio (S/N) evaluation and effectively addresses the limitations of conventional orthogonal testing, including the need for a large number of experimental runs and the occurrence of redundant procedures (Islam and Pramanik, 2016).

The experimental factors and levels were determined based on preliminary single-factor tests and analysis of variance (ANOVA), ensuring that the selected parameter ranges adequately represented the main factors influencing system performance. To assess the effects of different parameter levels on seed distribution uniformity and detection performance under high-throughput conditions, the following factors were included: seed socket arrangement, seed spreading roller speed, conveyor motor speed, seed box inclination, seed drop chute inclination, and conveyor belt type. The levels were defined as follows. The seed socket arrangement was classified as either deflected or non-deflected. The spreading roller speed was set to 50, 150, and 250 steps per second, and the conveyor motor speed was also set to 50, 150, and 250 steps per second. The seed box inclination angles were 20°, 30°, and 40°, while the chute inclination angles were 60°, 70°, and 80°. Three conveyor belt types were tested: a smooth white surface, a white diamond-patterned belt, and an enhanced diamond-patterned belt with a deflector partition at the end of the chute.

The Taguchi design is shown in **Table 1**. Each experiment was repeated three times across three groups: (1) imported soybeans only, (2) soybeans mixed with 1% *A. artemisiifolia* seeds, and (3) soybeans mixed with 1% *A. trifida* seeds. Each group produced a minimum of 100 seed images for analysis.

**Table 1:** Taguchi experimental setup.

Serial number	Forms of seed socket arrangement	Spreading roller speed	Conveyor motor speed	Seed box inclination	Seed drop chute inclination	Conveyor type
1	deflection	50	50	20	60	1
2	deflection	50	150	30	70	2
3	deflection	50	250	40	80	3
4	deflection	150	50	20	70	2
5	deflection	150	150	30	80	3
6	deflection	150	250	40	60	1
7	deflection	250	50	30	60	3
8	deflection	250	150	40	70	1
9	deflection	250	250	20	80	2
10	non-deflection	50	50	40	80	2
11	non-deflection	50	150	20	60	3
12	non-deflection	50	250	30	70	1
13	non-deflection	150	50	30	80	1
14	non-deflection	150	150	40	60	2
15	non-deflection	150	250	20	70	3
16	non-deflection	250	50	40	70	3
17	non-deflection	250	150	20	80	1
18	non-deflection	250	250	30	60	2

**Table 1** Taguchi experimental setup.

#### 2.4.2 Moran’s I coefficient

The intra-frame spatial uniformity was evaluated using Moran’s I coefficient, which ranges from −1 to 1, with values closer to 0 indicating greater uniformity. With a fixed weight matrix, this metric remains comparable across different resolutions and fields of view (Wang et al., 2023). Compared with the coefficient of variation, entropy, or nearest-neighbor index, Moran’s I more accurately captures local clustering effects and thus provides a robust quantitative assessment of seed distribution within the detection area. A value near 1 indicates seed clustering, a value near −1 suggests dispersion, and a value around 0 represents a random distribution, reflecting good uniformity. This metric effectively evaluates both the performance and the spatial uniformity of seed deployment by the detection device, thereby supporting subsequent optimization. Moran’s I was calculated using Equations 1 and Equations 2 (Wang et al., 2023).


(1) $$I=\frac{N\sum_{i}\sum_{j}w_{\text{ij}}(x_{\text{i}}-\bar{x})(x_{\text{j}}-\bar{x})}{S_{0}\sum_{i}{\left(x_{\text{i}}-\bar{x}\right)}^{2}}$$

(2) $$S_{0}=\sum_{i}\sum_{j}w_{\text{ij}}$$

where:

N——Total number of observations,

$x_{\text{i}}$ , $x_{\text{j}}$ ——Values of variables at positions i and j,

$\bar{x}$ ——Mean value of x,

$w_{\text{ij}}$ ——Spatial weights between positions i and j,

$S_{0}$ ——Sum of all spatial weights.

To compute Moran’s I coefficient for the original image, the proportion of target pixels within each local region of the binary map must first be obtained as baseline data. Accordingly, the original image was preprocessed to enable this analysis.

#### 2.4.3 Image processing for Moran’s I coefficient calculation

**Figure 8:**
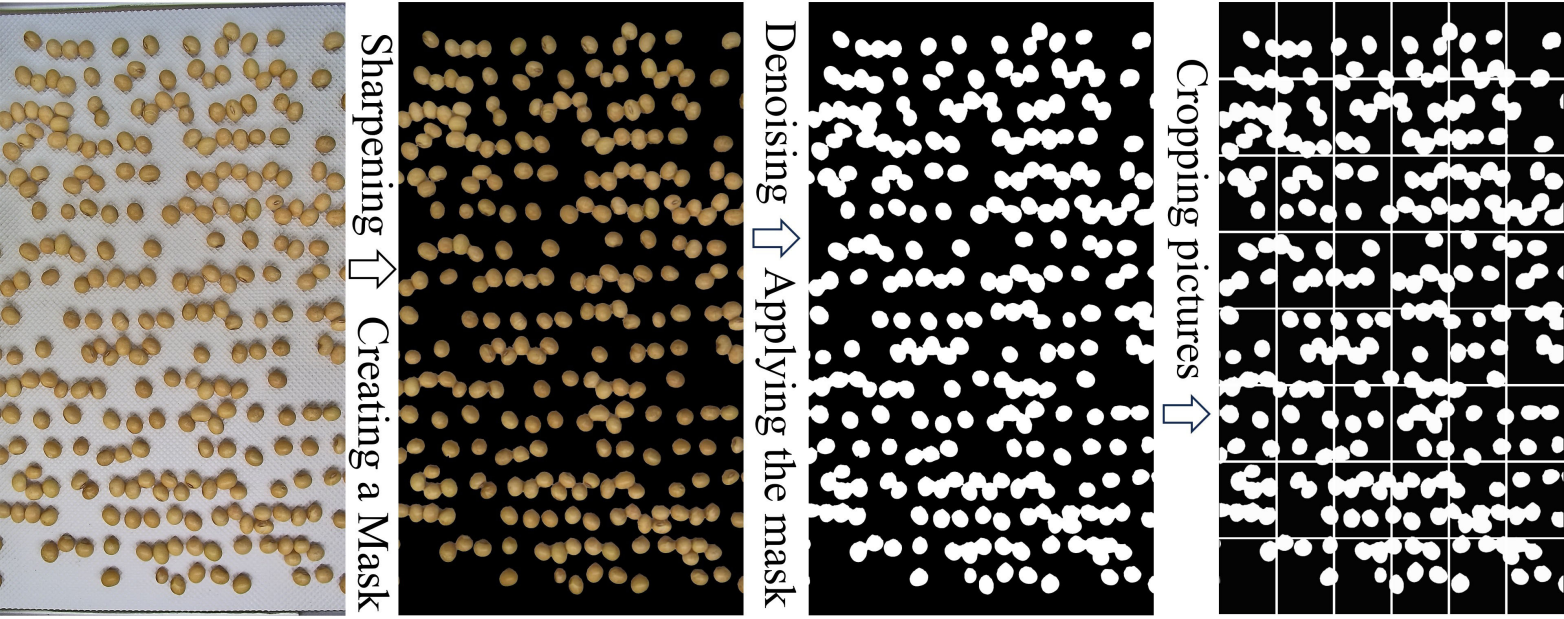
Image processing workflow.

Image processing was performed using MATLAB. The HSV color space was applied for seed segmentation. Initially, the original RGB image was sharpened using an unsharp mask to suppress noise. The denoised image was then converted into H, S, and V channels to extract the seed color, resulting in a binary image. To evaluate seed distribution, the binary image (3840 × 2160 pixels) was divided into 48 equally sized regions, each measuring 480 × 360 pixels. The percentage of seed pixels in each region was calculated as baseline data, which enabled the computation of Moran’s I coefficients for the original image using Equations 1 and 2. A schematic diagram of the image processing workflow is presented in **Figure 8**.

#### 2.4.4 Analysis of variance

ANOVA (Analysis of Variance) was conducted for both the mean and the signal-to-noise ratio (S/N). This statistical technique is widely used to assess the significance of main factor effects, allowing researchers to identify which variables should be retained in the predictive model and which can be considered statistically insignificant or attributed to random variation. The formula for calculating the sum of squares is presented in Equation 3 (Jadhav et al., 2024).


(3) $$SS=\frac{n}{l}\sum_{i=l}^{n}{\left(\bar{y_{\text{i}}}-\bar{y}\right)}^{2}$$

where n denotes the number of experimental repetitions; l represents the number of levels for the factor; $y_{\text{i}}$ is the mean value of the characteristic at the i level; and $\bar{Y}$ is the overall mean of the characteristic values.

The formula for calculating the degrees of freedom (DOF) is provided in Equation 4 (Jadhav et al., 2024).


(4) DOF=l−1

The formula for calculating the mean square (MS) is provided in Equation 5 (Jadhav et al., 2024).


(5) $$MS=\frac{SS}{DOF}$$

The F value is a statistical indicator used in ANOVA to evaluate the significance of differences between groups. It is calculated as the ratio of between-group variance to within-group variance (Thakuria and Erkinbaev, 2024). A higher F value indicates that the variation between groups is greater than the variation within groups.

The P value is used to determine the statistical significance of the F value. A commonly accepted threshold is 0.05. If the P value falls below this threshold, the null hypothesis is rejected, indicating that the differences among group means are statistically significant.

### 2.5 Detection method for three types of seed images

#### 2.5.1 Image dataset construction

All image data were collected directly from the single-seed uniform distribution and spreading device. No data augmentation was applied, as the dataset already included variations in seed density, overlap, and occlusion under stable illumination and background conditions. To maintain the validity of the Moran’s I–based uniformity evaluation, geometric and concatenation-type augmentations were excluded. In total, 5,567 images (3840 × 2160 pixels) were acquired. From these, 1,142 images representing different seed distribution patterns were manually annotated using the X-AnyLabeling tool. A subset of 228 images with varying densities was evenly allocated to the test set, and the remaining 914 images were split into training and validation sets at a 3:1 ratio.

#### 2.5.2 Training environment and methods

Model training was conducted on a Windows 10 workstation configured with 64 GB of RAM, an Intel Xeon Silver 4210 CPU, a Quadro RTX 4000 GPU, Python 3.9.18, CUDA 12.2, and PyTorch 1.10.1 with OpenCV 10.2 support. Stochastic Gradient Descent (SGD) was used as the optimizer. The initial learning rate was set to 0.01, the weight decay was 0.0005, the batch size was 8, and the training was conducted for 200 epochs.

#### 2.5.3 Evaluation indicators

Model performance was evaluated using Precision (P), Recall (R), and mean Average Precision (mAP). These metrics were calculated using Equations 6, Equation 7, and Equation 8 (Zhao et al., 2025).


(6) $$\text{Precision}=\frac{TP}{TP+FP}$$

(7) $$\text{Recall}=\frac{TP}{TP+FN}$$

(8) $$\text{AP}=\int_{0}^{1}p(r)dr$$

TP denotes true positives, FP denotes false positives, and FN denotes false negatives. TP refers to the number of correctly identified positive samples. FP represents the number of negative samples that were incorrectly labeled as positive, and FN refers to the number of positive samples that were incorrectly labeled as negative.

#### 2.5.4 YOLO_P2 model

To enable real-time detection of quarantine weed seeds and ensure compatibility with embedded deployment, several classic lightweight models were evaluated, including YOLOv5s, YOLOv7-tiny, and YOLOv8n. Among these models, YOLOv8n demonstrated the best detection performance, achieving the highest mean Average Precision (mAP), and was selected as the detection model for the system. However, among the three seed types, *A. artemisiifolia* exhibited the lowest detection accuracy. This is primarily due to its significantly smaller size compared to imported soybean and *A. trifida* seeds, which results in large target scale differences and reduced model performance.

**Figure 9:**
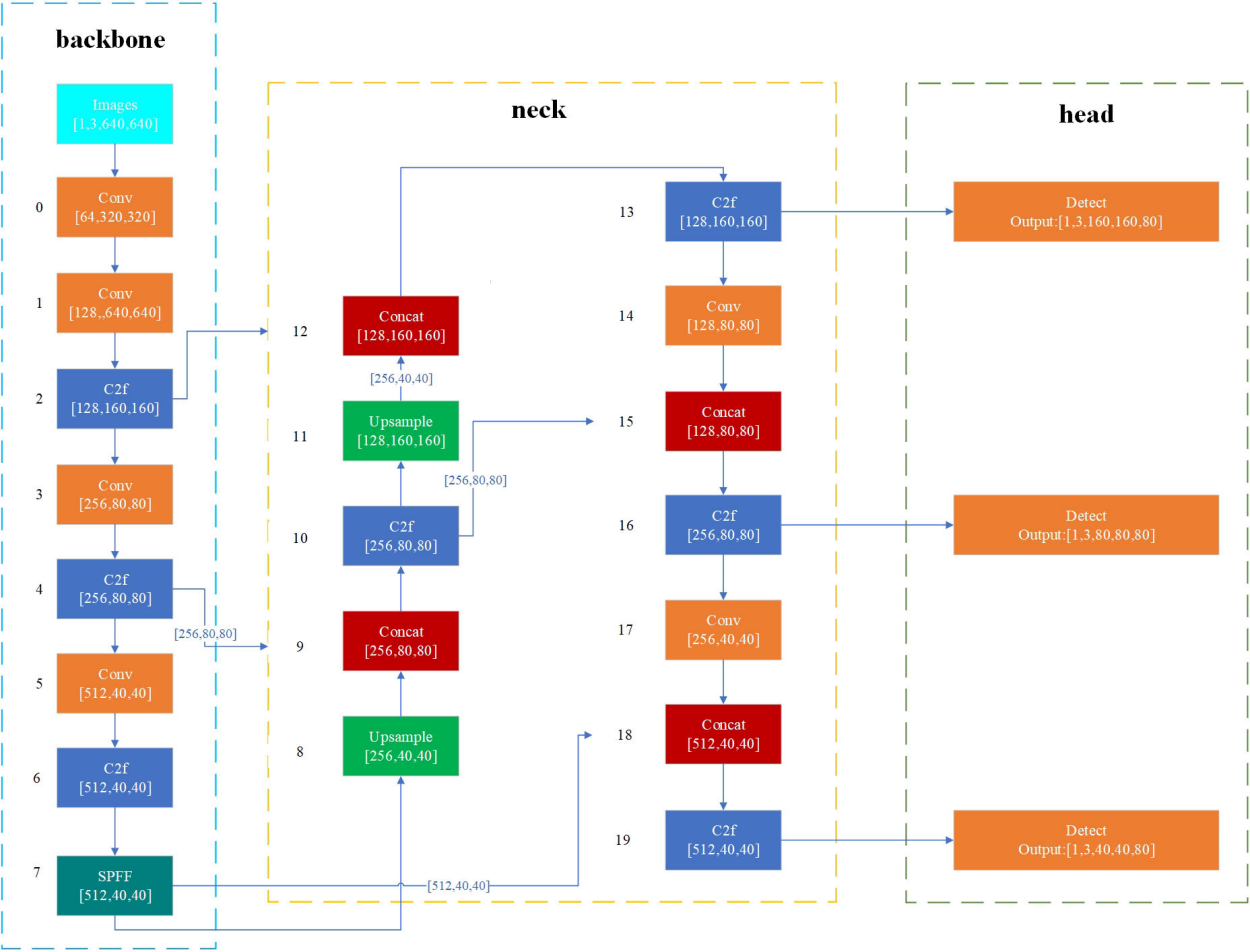
Structure of YOLO_P2.

To address this issue and improve detection accuracy for *A. artemisiifolia*, targeted modifications were applied to the YOLOv8n model. A P2 detection head was added to enhance the model’s ability to detect small objects. Because the inclusion of the P2 head increases model complexity, and all target objects in this application are relatively small with no large-scale instances, the P5 detection head was removed. The architecture of the improved YOLO_P2 model is illustrated in **Figure 9**.

### 2.6 Verification experiment

Based on the optimized parameter combination derived from the Taguchi experiment, the single-seed uniform distribution and spreading device was configured accordingly. After the successful deployment of the detection model and control system, validation experiments were conducted to evaluate the system’s performance.

The experiments simulated a customs quarantine scenario in accordance with the national entry and exit inspection and quarantine standard (SN/T 4979-2017). For each trial, 500 ± 10 g of imported soybean seeds were used. To replicate the natural distribution of quarantine weed seeds in imported soybeans, *A. trifida* and *A. artemisiifolia* seeds were introduced into the samples at a mass ratio of 1 ± 0.05% and a count ratio of 5 ± 0.5%. Specifically, 5 ± 1 g and 100 ± 10 seeds of *A. trifida*, along with 5 ± 1 g of *A. artemisiifolia*, were added to each 500 ± 10 g sample of soybeans (approximately 2,000 ± 40 seeds per sample).

This configuration ensured that the test conditions were both controllable and repeatable. The validation procedure was conducted across three independent groups to comprehensively assess the system’s detection performance.

## 3 Results

### 3.1 Results of the Taguchi experiment

**Figure 10:**
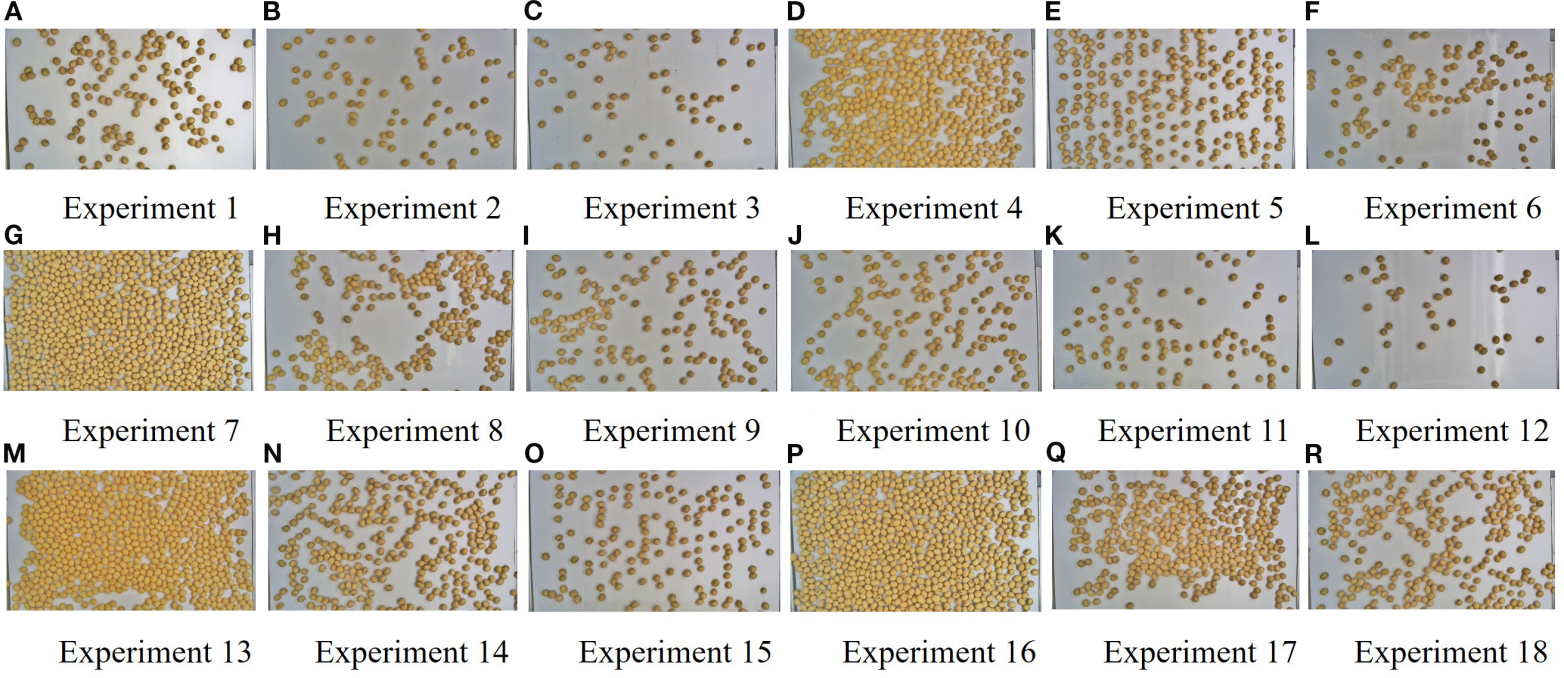
Effect of different parameters on the seed distribution.

A total of 5,567 images were collected throughout the experiment, with representative examples shown in **Figure 10**. These images reveal distinct variations in seed spreading performance across different experimental groups. Using images of imported soybeans as a reference, the seed distribution patterns ranged from sparse to dense and from disordered to well organized. This suggests that the parameter combinations of the device components had a significant influence on the uniformity and effectiveness of seed distribution.

Among all experiments, Groups 5 and 15 demonstrated the most effective seed spreading, with seeds uniformly distributed across the detection area and minimal occurrences of seed adhesion or occlusion. This determination was supported by quantitative results: the Moran’s I coefficients of Groups 5 and 15 were approximately 0.07 and 0.01, respectively, and both groups exhibited the lowest occlusion rates. Together, these metrics indicate superior spatial uniformity and visibility compared with the other groups. In contrast, Groups 4, 7, 13, and 16 exhibited notable seed clumping and shadowing, while Groups 2, 3, 6, 11, and 12 showed uneven or sparse seed distributions. To further evaluate these results, the images obtained from the Taguchi experiments were processed and analyzed using MATLAB to enable detailed quantitative assessment.

**Figure 11:**
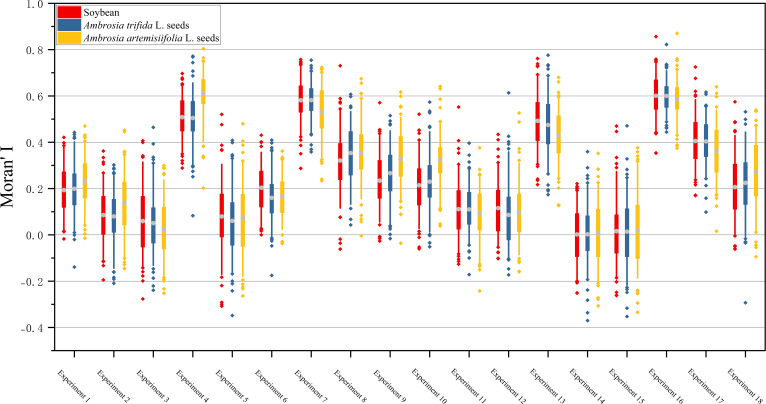
Box plots of Moran’s I coefficients for each experimental group.

Moran’s I coefficients for each image were calculated using MATLAB, and the resulting box plots for each experimental group are shown in **Figure 11**.

The mean Moran’s I coefficients across all groups were greater than zero, indicating an overall tendency toward clustered seed distribution. This result was consistent with the experimental design objective, which aimed to reduce seed adhesion and occlusion while maintaining a dense seed arrangement.

In most experimental groups, intragroup variation in Moran’s I values remained within 0.05. However, a noticeable difference of 0.1 was observed between Experiment 4 and Experiment 10. As shown in **Table 1**, both experiments used the same settings for conveyor motor speed (50 steps per second) and conveyor belt type (Type 3). This suggests that under these conditions, noise factors had a substantial impact on seed spreading performance, leading to inconsistent results.

Among all trials, Experiments 14 and 15 produced the most satisfactory outcomes, with mean Moran’s I values approaching zero, indicating highly uniform seed distribution. In contrast, Experiments 4, 7, 13, and 16 exhibited severe seed adhesion and occlusion, with significantly higher mean Moran’s I values. Experiments 1, 8, 9, 10, and 18 showed scattered or sparse seed distributions, with average Moran’s I values mostly exceeding 0.2.

### 3.2 Analysis of Taguchi experimental results

#### 3.2.1 Mean and S/N analysis

In the Taguchi method, quality characteristics are classified into two main categories: metrological characteristics and counting characteristics. Metrological characteristics are further divided into three types. These include those where a larger value is preferred, those where a smaller value is preferred, and those where the value should be as close as possible to a specific target. The signal-to-noise ratio (S/N) is used as a statistical indicator to evaluate the stability of these quality characteristics. For characteristics in which smaller values are preferred, the S/N ratio is calculated using Equation 9 (Islam and Pramanik, 2016).


(9) $$\frac{S}{N}=-10\log\frac{1}{m}\sum_{i=1}^{n}Y_{\text{i}}^{2}$$

where m is the number of observations and where $Y_{\text{i}}$ represents the observed values.

Section 3.1 presents the results of Moran’s I coefficient, where the average values obtained from the experiments were all greater than zero. The goal of this study is to achieve stable and uniform seed distribution by identifying the optimal combination of factor levels for the detection device, with a target Moran’s I coefficient close to zero. Accordingly, the signal-to-noise (S/N) ratio formula for the smaller-the-better characteristic was applied to analyze the experimental data. To evaluate the effects and significance of various factor levels on the performance of the single-seed uniform distribution and spreading device, both mean and S/N analyses were performed using Minitab.

**Table 2** presents the S/N ratios, mean values, and corresponding rankings of Moran’s I coefficients. A higher S/N ratio indicates more stable experimental outcomes. Regarding S/N ranking, the seed spreading roller speed had the greatest influence on performance, followed by the conveyor motor speed, conveyor type, seed box inclination, seed drop chute inclination, and the arrangement of seed sockets. In terms of mean value ranking, conveyor motor speed had the most substantial effect, followed by the seed spreading roller speed, seed drop chute inclination, conveyor type, seed box inclination, and socket arrangement.

**Table 2:** S/N and the mean of Moran’s I coefficient.

Level	Forms of seed socket arrangement		Seed spreading roller speed		Conveyor motor speed		Seed box inclination		Seed drop chute inclination		Conveyor belt type	
	S/n	Mean	S/n	Mean	S/n	Mean	S/n	Mean	S/n	Mean	S/n	Mean
1	14.366	0.2621	18.627	0.1389	7.636	0.4423	15.423	0.2622	18.921	0.2149	12.339	0.2765
2	18.508	0.2407	22.379	0.2102	21.201	0.1695	14.550	0.2539	15.649	0.2870	17.509	0.2491
3			8.260	0.4050	20.430	0.1424	19.294	0.2381	14.696	0.2523	19.418	0.2286
Ordinal rank	6	6	1	2	2	1	4	5	5	3	3	4

By comparing the rankings of the S/N ratios and the mean values, it is evident that seed spreading roller speed and conveyor motor speed are the most influential factors, as they consistently occupy the top two positions in both analyses. Therefore, when selecting the optimal levels for these two factors, it is important to consider both mean performance and stability, as reflected by the S/N ratio. The arrangement of seed sockets had the least impact, ranking sixth in both evaluations.

**Table 2** S/N and the mean of Moran’s I coefficient.

**Figure 12:**
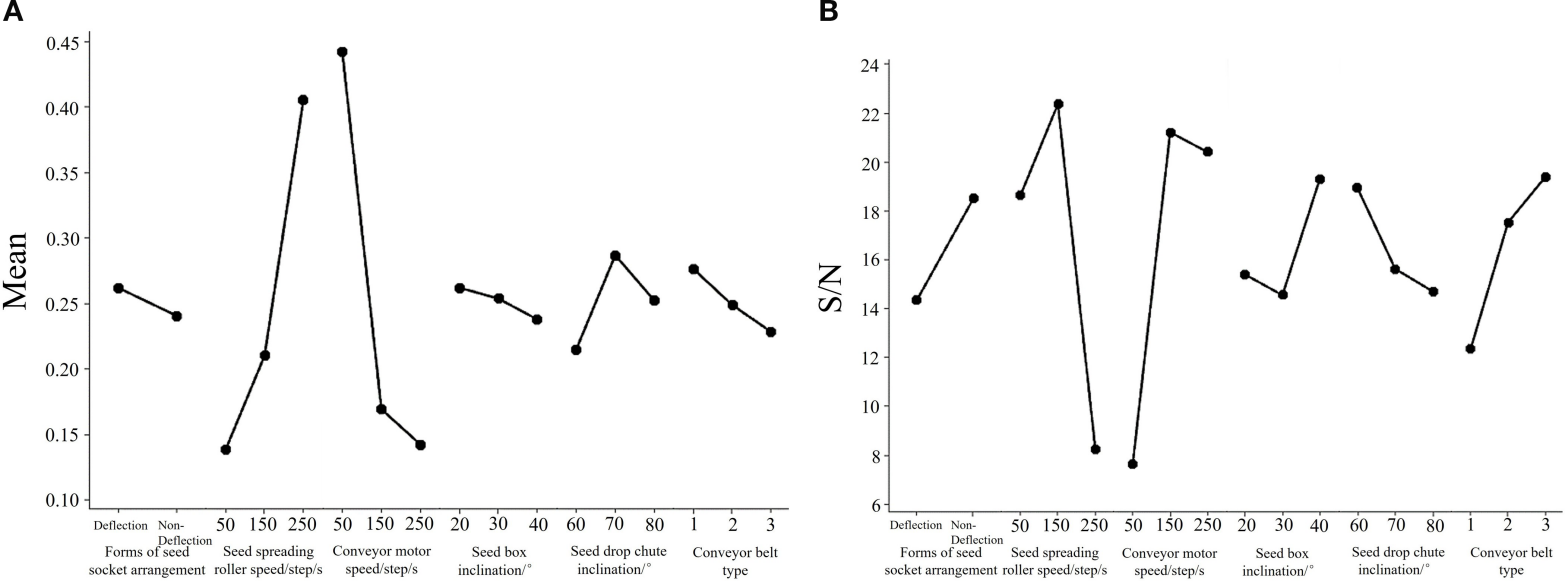
**(A)** Mean and **(B)** S/N plots of Moran’s I coefficient.

**Figure 12** presents the mean and signal-to-noise (S/N) plots of Moran’s I coefficient. According to the mean plot shown in **Figure 12a**, the optimal seed distribution, represented by the Moran’s I coefficient value closest to zero, was achieved under the following parameter settings: a seed socket arrangement without deflection, a seed spreading roller speed of 50 steps per second, a conveyor motor speed of 250 steps per second, a seed box inclination angle of 40°, a seed drop chute inclination angle of 60°, and conveyor belt type 3.

In comparison, the S/N plot shown in **Figure 12b** indicates that the most stable seed spreading results across repeated experiments were obtained with a configuration consisting of a seed socket arrangement without deflection, a roller speed of 150 steps per second, a conveyor motor speed of 150 steps per second, a seed box inclination of 40°, a drop chute inclination of 60°, and conveyor belt type 3. This configuration produced the highest signal-to-noise ratios.

#### 3.2.2 Analysis of variance

P values were derived from the degrees of freedom and the corresponding F values, as shown in **Table 3**. The analysis of the signal-to-noise (S/N) ratio revealed that both the seed spreading roller speed and the conveyor motor speed produced relatively high F values, although the difference between them was small. Since all P values exceeded the threshold of 0.05, none of the factors exhibited statistical significance in the S/N analysis. Therefore, the optimal combination of factor levels could not be determined based on the S/N results alone.

**Table 3:** ANOVA of the mean and S/N.

Source of variance	DOF		Seq SS		Adj SS		Adj MS		F		P	
	S/n	Mean	S/n	Mean	S/n	Mean	S/n	Mean	S/n	Mean	S/n	Mean
Forms of seed socket arrangement	1	1	78.32	0.002069	78.32	0.002069	78.32	0.002069	0.860	0.190	0.189	0.067
Seed spreading roller speed	2	2	641.74	0.227733	641.74	0.227733	320.87	0.113867	3.53	10.670	0.097	0.011
Conveyor motor speed	2	2	696.55	0.330180	696.55	0.330180	348.27	0.165090	3.83	15.470	0.085	0.004
Seed box inclination	2	2	76.49	0.001787	76.49	0.001787	38.25	0.000894	0.42	0.080	0.375	0.092
Seed drop chute inclination	2	2	58.93	0.015588	58.93	0.015588	29.46	0.007794	0.32	0.730	0.435	0.051
Conveyor belt type	2	2	160.96	0.006939	160.96	0.006939	80.48	0.003470	0.880	0.320	0.260	0.073

In contrast, the ANOVA of the mean values indicated significant effects for conveyor motor speed (P = 0.004) and seed spreading roller speed (P = 0.011), as their P values were below 0.05. When the results of both the mean and S/N analyses are considered together, seed spreading roller speed and conveyor motor speed are identified as the most influential factors affecting the uniformity of seed distribution. This finding is consistent with the rankings shown in **Table 2**. The conveyor belt type also showed a considerable effect, while the other factors had limited influence.

**Table 3** ANOVA of the mean and S/N.

In conclusion, the seed spreading roller speed and the conveyor motor speed are the principal factors influencing the performance of the single-seed uniform distribution and spreading system. The mean plot (**Figure 12a**) indicates that the Moran’s I coefficient is closest to zero when the roller speed is set to 50 steps per second and the conveyor motor speed is set to 250 steps per second. However, the S/N plot (**Figure 12b**) and the ANOVA results show that more stable outcomes are achieved when both speeds are set to 150 steps per second.

Considering the device’s dual requirements for precision and operational consistency, along with the minor differences observed in the mean plot and the improved detection performance shown in **Figure 9**, a speed of 150 steps per second was adopted as the optimal value for both the roller and the conveyor motor. Among the three conveyor belt types tested, type 3 yielded the most favorable results and was therefore selected. The remaining factors were found to have limited influence. As a result, the profiling holes were arranged in a deflected pattern, the seed box inclination was set to 40°, and the seed drop chute inclination was set to 60°. The complete optimized parameter configuration is summarized in **Table 4**.

**Table 4:** Optimal levels of detection device.

Evaluation indicators	Forms of seed socket arrangement	Spreading roller speed	Conveyor motor speed	Seed box inclination	Seed drop chute inclination	Conveyor type
Moran’s I coefficient	deflection	150 steps/s	150 steps/s	30°	70°	3

**Table 4** Optimal levels of detection device.

Following the optimization and physical assembly of the single-seed uniform distribution and spreading device, the next phase of the work focused on improving the deep learning model to address the relatively low detection accuracy associated with small targets, particularly *A. artemisiifolia* seeds.

### 3.3 Detection model and deployment

#### 3.3.1 Detection model

**Figure 13:**
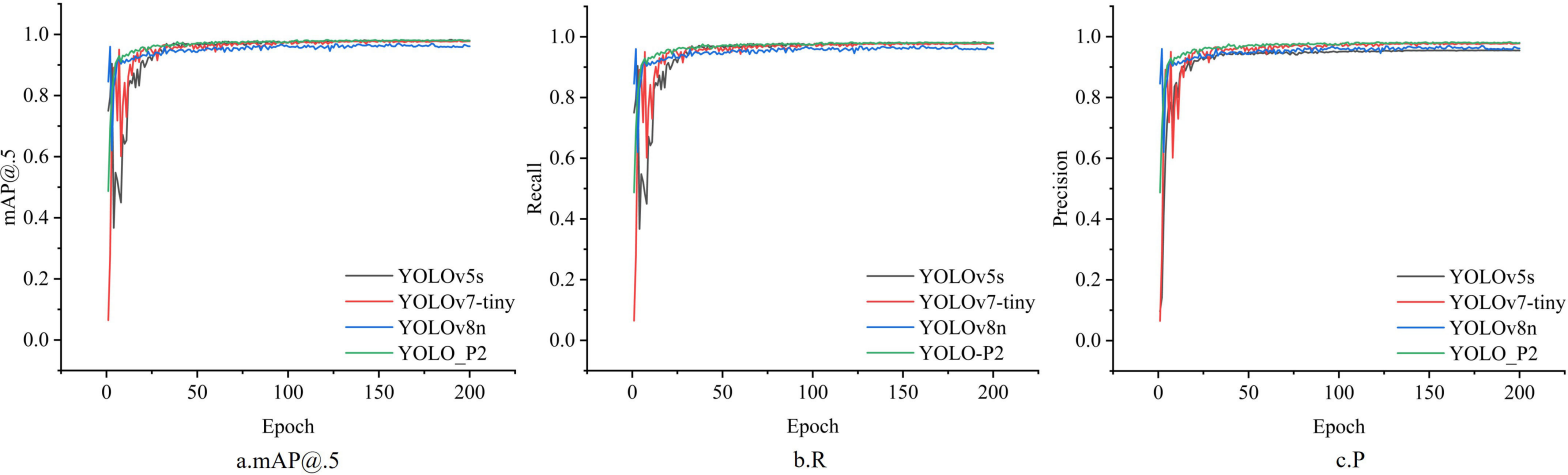
Plots of P, R and mAP variations for different models.

The training outcomes are summarized in **Table 5**, and the trends in mAP and Recall for each model are illustrated in **Figure 13**. As shown in **Figure 13**, the mAP, Precision, Recall, and corresponding curves for the YOLOv5s and YOLOv7-tiny models exhibit substantial fluctuations, indicating unstable detection performance. In contrast, the modified YOLO_P2 model demonstrates reduced fluctuations and improved stability.

**Table 5:** Comparative results of different models.

Model	Class	Precision	Recall	mAP	Params/m	Model size/MB	Epoch
YOLOv5s	all	0.982	0.932	0.951	7.2	14	200
	soybean	0.988	0.943	0.953			
	*A. trifida* seeds	0.986	0.992	0.996			
	*A. artemisiifolia* seeds	0.972	0.861	0.904			
YOLOv7-tiny	all	0.977	0.936	0.946	6.2	12	
	soybean	0.982	0.945	0.954			
	*A. trifida* seeds	0.986	0.993	0.997			
	*A. artemisiifolia* seeds	0.964	0.87	0.88			
YOLOv8n	all	0.96	0.914	0.947	3.2	6.2	
	soybean	0.98	0.944	0.966			
	*A. trifida* seeds	0.98	0.996	0.995			
	*A. artemisiifolia* seeds	0.919	0.802	0.88			
YOLO_P2	all	0.983	0.945	0.961	3.3	6.3	
	soybean	0.987	0.939	0.963			
	*A. trifida* seeds	0.99	0.994	0.995			
	*A. artemisiifolia* seeds	0.972	0.901	0.926			

The average mAP across all three models was approximately 95%. The YOLOv5s model achieved the highest detection accuracy for *A. artemisiifolia* seeds at 90.4%, which was 2.4 percentage points higher than those achieved by the other two models. For imported soybeans, YOLOv8n attained the best detection accuracy at 96.6%, outperforming YOLOv5s and YOLOv7-tiny by 1.3 and 1.2 percentage points, respectively. Notably, YOLO_P2, built upon YOLOv8n, significantly enhanced the detection of small targets (such as *A. artemisiifolia* seeds) without increasing model parameters or model size, thereby achieving superior performance while maintaining efficiency.

#### 3.3.2 Model deployment

**Figure 14:**
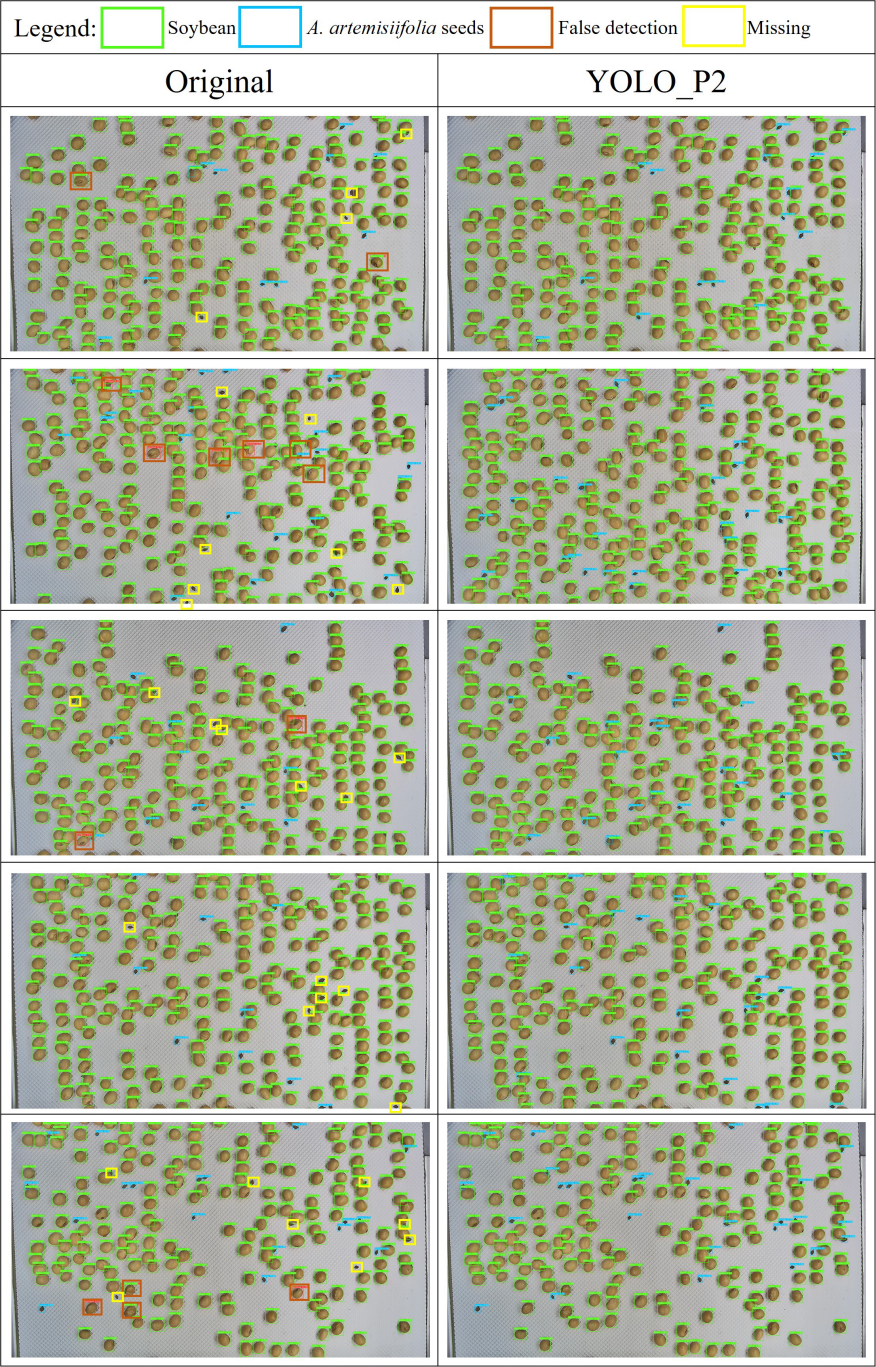
Detection performance for *A. artemisiifolia* seeds using the original model and the YOLO_P2 model.

As illustrated in **Figure 14**, both the original model (YOLOv8n) and the modified YOLO_P2 model were deployed on an embedded platform for testing and comparison. The platform used was the NVIDIA Jetson Orin NX 16 GB, configured with Jetpack 5.1, CUDA version 11.4.315, cuDNN version 8.6.0.166, Torch version 2.0.0+nv23.5, and Torchvision version 0.15.1.

The original model processed a single image in 57.8 milliseconds, consisting of 7.5 milliseconds for preprocessing, 31.1 milliseconds for inference, and 19.2 milliseconds for postprocessing. In comparison, the YOLO_P2 model required 64.2 milliseconds to process a single image, including 7.6 milliseconds for preprocessing, 38.0 milliseconds for inference, and 18.6 milliseconds for postprocessing. The improved model resulted in an increase in total processing time of only 6.4 milliseconds.

**Figure 14** presents a comparison between the original model and the YOLO_P2 model in detecting *A. artemisiifolia* seeds. The original model exhibited recognition errors and missed detections, particularly for the smaller-sized *A. artemisiifolia* seeds. Its performance was notably poor when these seeds were partially obscured by soybean seeds. In contrast, the YOLO_P2 model effectively addressed these issues and demonstrated significantly improved performance, especially in detecting *A. artemisiifolia* seeds under occlusion.

**Figure 15:**
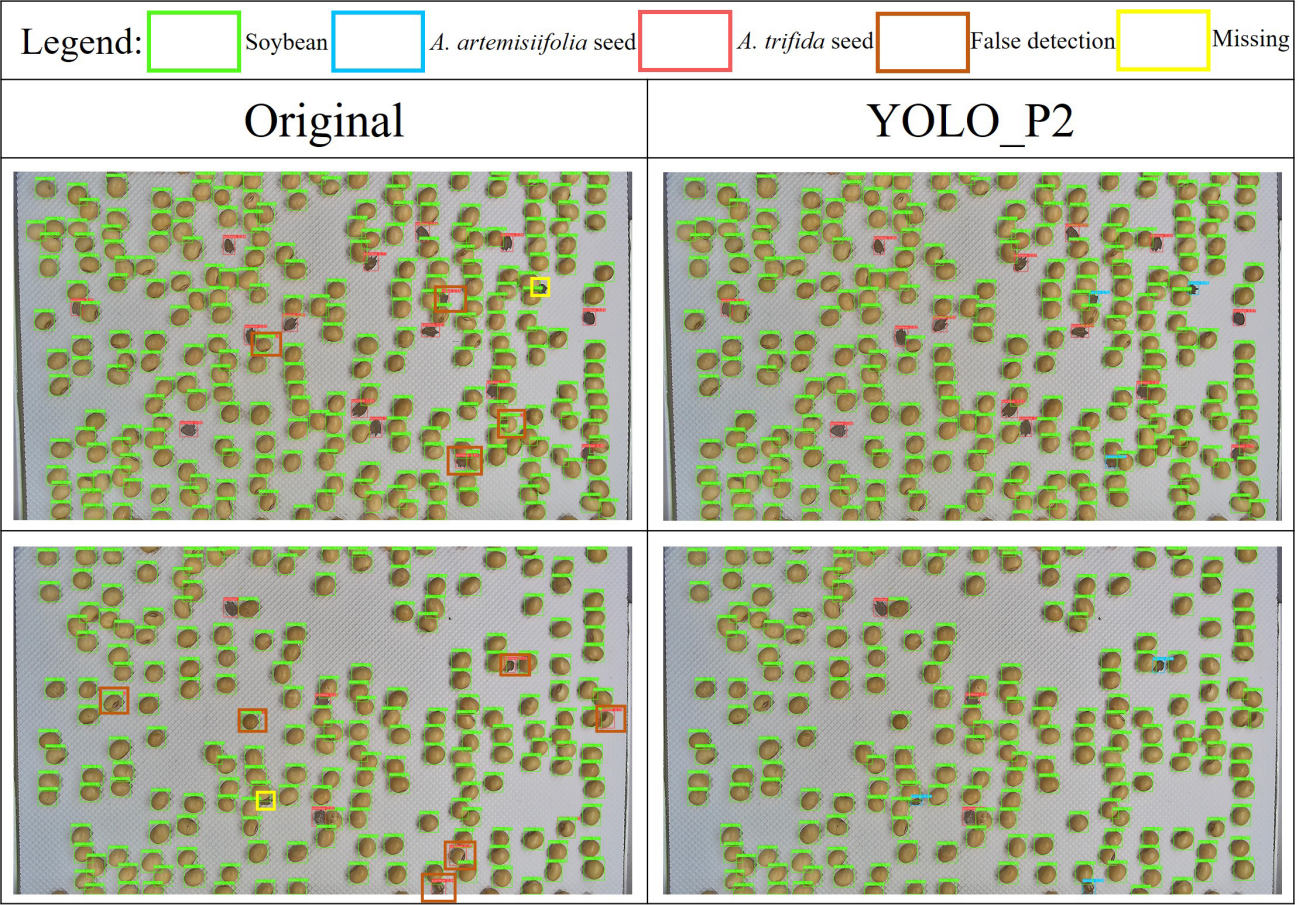
Detection performance for mixed samples containing imported soybeans, *A. trifida* L., and *A. artemisiifolia* L. seeds.

**Figure 15** presents a comparison between the original model and the YOLO_P2 model in detecting mixed samples containing imported soybeans, *A. trifida*, and *A. artemisiifolia.* seeds. The original model exhibited both false detections and missed detections during the recognition process, particularly showing poor performance when the *A. artemisiifolia* seeds were partially occluded. In contrast, the YOLO_P2 model demonstrated superior performance in handling occlusion and accurately identifying *A. artemisiifolia* seeds, resulting in a notable improvement in overall detection accuracy.

### 3.4 Verification experiment

The experimental outcomes for *A. trifida* and *A. artemisiifolia.* seeds are presented in **Tables 6** and **7**, respectively. Following this, the three types of seeds were mixed, and a series of validation experiments was conducted. The results obtained from these mixed-seed trials are summarized in **Table 8**.

**Table 6:** Verification experiment results for *A. trifida* seeds.

Serial number	No. of imported soybeans	No. of imported soybeans detected	Detecting accuracy of imported soybeans/%	No. of *A. trifida* seeds	No. of *A. trifida* seeds detected	Percentage of *A. trifida* seeds/%	Detecting the accuracy of *A. trifida* seeds/%	Leakage rate/%	Detection time/min
1	1937	1898	98.17	97	93	4.89	96.39	3.61	7.2
2	2016	1976	98.11	103	98	4.96	96.47	3.53	6.8
3	1984	1944	98.04	99	95	4.89	96.24	3.76	7.1
Mean	1979	1939	98.11	99	95	4.91	96.37	3.63	7.0

**Table 7:** Verification experiment results for *A. artemisiifolia* seeds.

Serial number	No. of imported soybeans	No. of imported soybeans detected	Detecting accuracy of imported soybeans/%	No. of *A. artemisiifolia* seeds	No. of *A. artemisiifolia* seeds detected	Percentage of *A. artemisiifolia* seeds/%	Detecting the accuracy of *A. artemisiifolia* seeds/%	Leakage rate/%	Detection time/min
1	1976	1936	98.34	105	99	5.11	95.34	4.66	7.1
2	2009	1968	98.95	107	101	5.13	95.86	4.14	6.9
3	1994	1954	98.27	94	89	4.55	95.07	4.93	6.8
Mean	1993	1953	98.52	102	96	4.93	95.42	4.58	6.9

**Table 8:** Verification experiment results for three types of seeds.

Serial number	No. of imported soybeans	No. of imported soybeans detected	Detecting accuracy of imported soybeans/%	No. of *A. artemisiifolia* seeds	No. of *A. artemisiifolia* seeds detected	Detecting the accuracy of *A. artemisiifolia* seeds/%	No. of *A. trifida* seeds	No. of *A. trifida* seeds detected	Detecting the accuracy of *A. trifida* seeds/%	Average accuracy/%	Detection time/min
1	1954	1919	97.21	105	100	94.23	97	93	95.45	95.63	7.5
2	1997	1970	97.64	107	102	94.77	103	99	95.31	95.91	7.4
3	2014	1977	97.16	94	90	94.39	99	96	95.73	95.76	7.9
Mean	2003	1969	97.32	102	97	94.41	99	96	95.73	95.77	7.6

Regarding the detection of *A. trifida* seeds, the accuracy rates were recorded as 96.39%, 96.47%, and 96.24%, with an average of 96.37%. The corresponding miss rates were 3.61%, 3.53%, and 3.76%, resulting in an average of 3.63%. These results indicate that although *A. trifida* seeds represent a relatively small proportion of the sample, the system is capable of reliably identifying and accurately detecting the majority of these seeds. The observed miss rates remained within an acceptable range.

For *A. artemisiifolia.* seeds, the detection accuracy was slightly lower, with values of 95.34%, 95.86%, and 95.07%, yielding an average of 95.42%. The corresponding miss rates were 4.66%, 4.14%, and 4.93%, with an average of 4.58%. Although the accuracy was marginally lower than that for *A. trifida*, the system still exhibited relatively strong recognition capability. The slightly elevated miss rate may be attributed to factors such as the visual characteristics of *A. artemisiifolia.* seeds, their morphological similarity to soybeans, and the image quality captured by the camera. These findings suggest that future improvements in the detection of *A. artemisiifolia* seeds could be achieved by enhancing the algorithm or optimizing camera parameters to improve recognition precision.

In terms of detection time, each group of tests was completed within 6.8 to 7.9 minutes, all of which were shorter than the time required for manual inspection. This demonstrates the system’s ability to maintain stable operational efficiency under varying experimental conditions. Although minor differences in detection time were observed across different groups, the overall processing times remained consistent and met the experimental requirements.

During the detection of the three types of seeds, a slight decrease in accuracy and a slight increase in detection time were observed. However, both remained within the acceptable range defined by the system’s performance standards.

## 4 Discussion

This study introduces a reliable detection device for the quarantine inspection of imported soybeans at customs, specifically designed to identify two types of quarantine weed seeds, *A. trifida* and *A. artemisiifolia*. Although the focus is limited to these two species, they are among the most frequently intercepted in imported soybean shipments. The proposed detection system addresses a critical gap in the current development of quarantine detection equipment and provides both a novel approach and a valuable technical reference. The single-seed uniform distribution and spreading device, which incorporates parabolic seed sockets, offers notable advantages compared to conventional detection systems. It is capable of evenly and neatly distributing multiple seed types with distinct size differences and supports high-throughput image dataset acquisition. When integrated with the real-time detection system, it demonstrates high accuracy and satisfactory computational efficiency, thereby fulfilling the operational requirements of customs quarantine inspections.

In comparison with related approaches (Zhao et al., 2021; Wang et al., 2023), the proposed system demonstrates advantages in four key aspects: cost-effectiveness (hardware cost approximately ¥1500–2000 with a power consumption of ~15 W), processing speed (20–30 seeds per second), cross-seed adaptability (extension to similar small-seed species achievable through replacement of the seed dispenser and limited retraining), and optimization for customs applications (including batch traceability, automatic reinspection of anomalies, and self-recovery from clogging events). These features highlight the practical relevance of the system in real-world quarantine inspection scenarios. From an engineering perspective, challenges remain in ensuring robustness across varieties, maintaining long-term stability, facilitating convenient maintenance, and integrating seamlessly into inspection workflows.

Nonetheless, some limitations persist. The use of parabolic seed sockets has been associated with occasional seed jamming, which may hinder overall seeding efficiency. Future studies should aim to optimize the design of the profiling holes and improve the seed-clearing mechanism in order to minimize jamming and enhance throughput. While such modifications may increase structural complexity and affect the stability of the device, they remain a practical and worthwhile direction for further development. In addition, while the system shows detection potential for small-sized weed seeds within samples exhibiting substantial particle-size differences, its applicability to specific species or extreme morphological cases still requires validation through expanded data collection and model retraining. This adjustment provides a more accurate reflection of the system’s practical boundary conditions and avoids overgeneralisation of its applicability. Effectively addressing these challenges is expected to enhance the generalisability and practical applicability of equipment for quarantine weed seed detection.

## 5 Conclusion

In this study, a real-time, high-throughput, and cost-effective detection system for identifying quarantine weed seeds in imported soybeans was successfully developed and validated. To address the challenges of seed occlusion and uneven distribution in complex sample environments, a seed dispenser incorporating parabolic seed sockets was designed. This configuration enabled the uniform and orderly distribution of multiple seed types within a single image, maximizing the number of seeds per frame while effectively preventing mutual occlusion.

Furthermore, to improve distribution uniformity, the key structural parameters of the detection device were systematically optimized using the Taguchi experimental design method. This process led to the identification of an optimal parameter configuration. In addition, to satisfy the requirements for real-time detection and to enhance the recognition of small targets such as *A. artemisiifolia* seeds, the YOLO_P2 model was specifically modified. These targeted improvements significantly enhanced the detection performance of the model.

Verification experiments confirmed that the average detection accuracy reached 95.77%, with an average detection time of 7.6 minutes. The proposed detection system is suitable not only for the quarantine inspection of imported soybeans, but also for the identification of other seed types. Beyond its demonstrated performance on *A. artemisiifolia* and *A. trifida* seeds, the proposed model also exhibits strong transferability. With minimal additional data annotation and model fine-tuning, it can be extended to other small-sized seeds with similar particle size or morphology while maintaining the same hardware platform. This highlights the system’s reusability and scalability, and suggests promising potential for broader applications in quarantine inspection scenarios. This work offers an effective method for the rapid acquisition of high-quality, high-throughput seed images and may serve as a valuable tool for laboratory research and other applications requiring efficient generation of annotated datasets.

## Data Availability

The original contributions presented in the study are included in the article/supplementary material. Further inquiries can be directed to the corresponding author.

## References

[B1] Ali T. Zhou B. Cleary D. Xie W. (2022). The impact of climate change on China and Brazil’s soybean trade. Land 11, 2286. doi: 10.3390/land11122286

[B2] Baider C. Florens F. V. (2011). Control of invasive alien weeds averts imminent plant extinction. Biol. Invasions 13, 2641–2646. doi: 10.1007/s10530-011-9980-3

[B3] Cao M. Cao C. Zhang T. Guo W. (2025). A dielectric method for predicting pear firmness combining deep data augmentation and ensemble learning. Food Control 167, 110790. doi: 10.1016/j.foodcont.2024.110790

[B4] Chen C. Chen W. Zhang X. Qiu M. Jin B. He J.. (2025). A scene-adaptive reseeding system with missed seeding detection for double-disc air-suction seed meter based on an improved YOLOv8n algorithm. Comput. Electron. Agric. 237, 110682. doi: 10.1016/j.compag.2025.110682

[B5] Davies K. W. Sheley R. L. (2007). A conceptual framework for preventing the spatial dispersal of invasive plants. Weed Sci. 55, 178–184. doi: 10.1614/WS-06-161

[B6] de Lima D. P. Fioriolli J. C. Padula A. D. Pumi G. (2018). The impact of Chinese imports of soybean on port infrastructure in Brazil: a study based on the concept of the “Bullwhip Effect. J. Commod. Mark. 9, 55–76. doi: 10.1016/j.jcomm.2017.11.001

[B7] Diagne C. Leroy B. Vaissière A. C. Gozlan R. E. Roiz D. Jarić I.. (2021). High and rising economic costs of biological invasions worldwide. Nature 592, 571–576. doi: 10.1038/s41586-021-03405-6, PMID: 33790468

[B8] ElMasry G. Mandour N. Wagner M. H. Demilly D. Verdier J. Belin E.. (2019). Utilization of computer vision and multispectral imaging techniques for classification of cowpea (Vigna unguiculata) seeds. Plant Methods 15, 1–16. doi: 10.1186/s13007-019-0411-2, PMID: 30911323 PMC6417027

[B9] Hall C. M. (2011). Biosecurity, tourism and mobility: institutional arrangements for managing tourism-related biological invasions. J. Policy Res. Tour. Leis. Events 3, 256–280. doi: 10.1080/19407963.2011.576868

[B10] Huang M. Wang Q. Zhu Q. Qin J. Huang G. (2015). Review of seed quality and safety tests using optical sensing technologies. Seed Sci. Technol. 43, 337–366. doi: 10.15258/sst.2015.43.3.16

[B11] Íñiguez R. Gutiérrez S. Poblete-Echeverría C. Hernández I. Barrio I. Tardáguila J. (2024). Deep learning modelling for non-invasive grape bunch detection under diverse occlusion conditions. Comput. Electron. Agric. 226, 109421. doi: 10.1016/j.compag.2024.109421

[B12] Islam M. N. Pramanik A. (2016). Comparison of design of experiments via traditional and Taguchi method. J. Adv. Manuf. Syst. 15, 151–160. doi: 10.1142/S0219686716500116

[B13] Jadhav P. K. Sahai R. S. N. Solanke S. Gawande S. H. (2024). Multi-objective optimization of EN19 steel milling parameters using Taguchi, ANOVA, and TOPSIS approaches. J. Alloys Metall. Syst. 7, 100102. doi: 10.1016/j.jalmes.2024.100102

[B14] Jay M. Morad M. Bell A. (2003). Biosecurity, a policy dilemma for New Zealand. Land Use Policy 20, 121–129. doi: 10.1016/S0264-8377(03)00008-5

[B15] Lei R. Yan Z. Hu F. Zhu S. Xiong Y. Fan X. (2017). Rapid identification of quarantine invasive Solanum elaeagnifolium by real-time, isothermal recombinase polymerase amplification assay. RSC Adv. 7, 52573–52580. doi: 10.1039/c7ra10781a

[B16] Liu Z. Abeyrathna R. R. D. Sampurno R. M. Nakaguchi V. M. Ahamed T. (2024). Faster-YOLO-AP: a lightweight apple detection algorithm based on improved YOLOv8 with a new efficient PDWConv in orchard. Comput. Electron. Agric. 223, 109118. doi: 10.1016/j.compag.2024.109118

[B17] Ma B. Hua Z. Wen Y. Deng H. Zhao Y. Pu L.. (2024). Using an improved lightweight YOLOv8 model for real-time detection of multi-stage apple fruit in complex orchard environments. Artif. Intell. Agric. 11, 70–82. doi: 10.1016/j.aiia.2024.02.001

[B18] Magarey R. D. Colunga-Garcia M. Fieselmann D. A. (2009). Plant biosecurity in the United States: roles, responsibilities, and information needs. BioScience 59, 875–884. doi: 10.1525/bio.2009.59.10.9

[B19] Meyerson L. A. Reaser J. K. (2002). Biosecurity: moving toward a comprehensive approach. BioScience 52, 593–600. doi: 10.1641/0006-3568(2002)052[0593:BMTACA]2.0.CO;2

[B20] Rahman A. Cho B. K. (2016). Assessment of seed quality using non-destructive measurement techniques: a review. Seed Sci. Res. 26, 285–305. doi: 10.1017/S0960258516000234

[B21] Thakuria A. Erkinbaev C. (2024). Real-time canola damage detection: an end-to-end framework with semi-automatic crusher and lightweight shuffleNetV2_YOLOv5s. Smart Agric. Technol. 7, 100399. doi: 10.1016/j.atech.2024.100399

[B22] Vega−Castellote M. Sánchez M. T. Kim M. S. Hwang C. Pérez−Marín D. (2025). Investigating the detection of peanuts in chopped nut products using hyperspectral imaging systems. J. Food Eng. 388, 112378. doi: 10.1016/j.jfoodeng.2024.112378

[B23] Wang Y. Lv W. Wang M. Chen X. Li Y. (2023). Application of improved Moran’s I in the evaluation of urban spatial development. Spat. Stat. 54, 100736. doi: 10.1016/j.spasta.2023.100736

[B24] Wang Q. Yu C. Zhang H. Chen Y. Liu C. (2023). Design and experiment of online cottonseed quality sorting device. Comput. Electron. Agric. 210, 107870. doi: 10.1016/j.compag.2023.107870

[B25] Whitehurst L. E. Cunard C. E. Reed J. N. Worthy S. J. Marsico T. D. Lucardi R. D.. (2020). Preliminary application of DNA barcoding toward the detection of viable plant propagules at an initial, international point-of-entry in Georgia, USA. Biol. Invasions 22, 1585–1606. doi: 10.1007/s10530-020-02204-w

[B26] Zhang Z. Guo H. Zhang Y. Ke Z. Guo Y. Sun K.. (2025). Towards accurate field counting of small pests with visual prompts. Comput. Electron. Agric. 237, 110635. doi: 10.1016/j.compag.2025.110635

[B27] Zhao G. Quan L. Li H. Feng H. Li S. Zhang S.. (2021). Real-time recognition system of soybean seed full-surface defects based on deep learning. Comput. Electron. Agric. 187, 106230. doi: 10.1016/j.compag.2021.106230

[B28] Zhao P. Wu X. Cheng H. Gao X. Liu Z. Lai Q.. (2025). Performance evaluation of a high-speed maize seed-metering device using an improved YOLOv5s object detection and tracking algorithm. Smart Agric. Technol., 11 10099. doi: 10.1016/j.atech.2025.100997

[B29] Zheng J. Wang X. Shi Y. Zhang X. Wu Y. Wang D.. (2024). Keypoint detection and diameter estimation of cabbage (Brassica oleracea L.) heads under varying occlusion degrees via YOLOv8n-CK network. Comput. Electron. Agric. 226, 109428. doi: 10.1016/j.compag.2024.109428

